# Editorial comment to the manuscript “Minimal invasive approach of post‐MI ventricular septal defect repair and coronary artery revascularization via left anterior minithoracotomy”

**DOI:** 10.1111/jocs.16015

**Published:** 2021-09-21

**Authors:** Marko Turina

**Affiliations:** ^1^ University of Zurich Zurich Switzerland

**Keywords:** artery, minithoracotomy

Authors are to be congratulated for their successful minimally invasive treatment of the infarction VSD via left anterior minithoracotomy, coupled with LIMA grafting to the LAD. It should be stressed that authors encountered an “optimal” infarction VSD, in an obvious subacute stage, with haemodynamic stabilization after 3 days of drug treatment. Such a stabilization period is hardly possible in an acute infarction VSD, which quickly progresses to a profound heart failure without surgical closure.^1^ But readers should credit the authors with a remarkable surgical dexterity, first for their dealing with the cannulation, administration of cardioplegia, and controlling pulmonary venous return when working only from the small anterior thoracotomy; and for their decision to harvest LIMA through the anterior thoracotomy. This approach makes LIMA harvesting much simpler^2^ and is used by many surgeons in minimally invasive off‐pump CABG.
